# Practical Observations on the Spasmodic Cholera of India

**Published:** 1831-04-01

**Authors:** 


### VIII.

### A FEW PRACTICAL OBSERVATIONS ON THE SPASMODIC CHOLERA OF INDIA. By *Alexander Smith*, M.D. Member of the Royal Asiatic Society, of the Asiatic Medical and Physical Societies of Calcutta, Assistant Surgeon of the King's Dragoon Guards.

THE epidemic form of spasmodic cholera, for more than thirteen years the fatal scourge of Hindoostan, has been practically known to the inhabitants of Europe only by the occasional appearance of a disease, which, in some of its leading symptoms, has borne a resemblance to the appalling coup de mort of India. During the present year, however, the rapid and deadly progress of an analogous distemper, over a great portion of the Russian Empire, renders the cholera an object of dread and interest to Europeans generally, and calls for every mite of information relative to the phenomena of this justly-termed inscrutable and intractable disease, which research can discover or experience supply. The author is, therefore, induced to offer the following observations, made during a period of severe suffering from this malady, and as containing the result of considerable practice.

### FIRST OBSERVATION.

*On the general Cause and Effects of Spasmodic Cholera.*—The cholera derives its exciting cause from a vitiated state of the atmosphere, which contaminates the blood during its circulation through the lungs of persons predisposed to the disease by the depressing passions, irregularity of the natural functions, or any debilitating power. The immediate effect of this contamination is, that a stream of deteriorated, probably decomposed blood, is thrown through the heart, into the vessels where the crassamentum becomes congested, and incapable of exciting or supporting the healthy organic action, while the vessels surcharged with the separated serum pours it into the stomach, intestines, and over the surface of the body. The absorbent function is suspended, the nerves fall into irregular action, the great lymphatic loses its influence over the organs which it supplies; the heart assumes a morbid imbecile excitement; the stomach falls into a state of violent inverted action, the hepatic and renal ducts are constricted; the intestines are incapable of freeing themselves from the morbid secretions which oppress them; the muscular energies are universally prostrated; and nothing escapes the shock save the sensorium, which continues sound amidst the agonizing spasm of its nerves, until the destruction of almost every other function, when worn out by intensity of suffering the mind also sinks into insensibility and yields to its fate.

### SECOND OBSERVATION.

*On the Predisposing Cause of Spasmodic Cholera.* Perhaps there exists no proof that any particular conformation of body, or any age, or occupation, or mode of living, have a direct tendency to produce a predisposition for this disease. Some well-marked cases have, however, occurred to the author, in which general exhaustion, shrunken features, and hollow, dark-encircled eyes, seemed to be induced by great anxiety and fatigue during very hot weather, and these cases terminated in collapsed cholera.

It is also worthy of remark, that of two corps, one was stationed during a late hot season, in crowded barracks, having a very low site, with a nulla or stagnant pool in the rear; but the men being cheerful and contented, cholera did not make its appearance among them—while the other corps, although cantoned in excellent detached barracks, situate on one of the most elevated and most healthy plains of central India, during the same season and within a hundred miles of the former regiment, being less happy and contented, were subjected to four visitations of the disease during the season, and lost a considerable number of men.

It may be added here that, in a majority of the fatal cases of cholera, potatoes, in an undigested state, were found in the stomach.

### THIRD OBSERVATION.

*On the Nature of the Miasm which excites Spasmodic Cholera.*—We have to regret that neither theory nor experiment have yet demonstrated, what the atmospheric contamination, which, in passing over certain districts, excites spasmodic cholera in predisposed persons, actually consists of; but we do know, from its effects, that the air bears along with it either combined or suspended, sometimes a very considerable proportion of the prostrating power, so that the number attacked with the worst form of the disease becomes at once great; and, at other times, that the atmosphere seems to be impregnated with so minute a portion of the miasm, that a mild form of the disease shews itself, and soon passes away. Spasmodic cholera often continues its havoc while a certain wind prevails, or during a particular state of the weather, and ceases when the wind or weather undergo any change. It is most virulent during the hot season of the year, and exercises its influence under the most opposite extremes of dryness and humidity of the atmosphere. Thus we have seen it disappear, when the rains were succeeded by warm, dry weather; we have known it disappear, also, when an easterly breeze interrupted the course of the hot, dry north-west winds; and, again, it has been dispelled by the first showers of the rainy season.

### Fourth Observation.

*On the Non-contagious Nature of Spasmodic Cholera.*—The Indian cholera possesses none of the characteristics which distinguish the contagious or infectious diseases; on the contrary, it appears to sweep along the surface of the earth, attacking the rich man in his insulated palace, and the poor in his lonely hut; the robust European and the effeminate Hindoo, wherever it finds either incapable of resisting its prostrating power. It is not confined to cities or to camps, it appears suddenly, remains until disturbed by some new motion in the atmosphere, and then vanishes without leaving the power in its victims to communicate any form of disease to their fellow-creatures. No instance has come under the observation of the author, where the diseased person infected another, and the immunity of the attendants, as well as of the friends of the sick, who often crowd around the death-bed of their unfortunate comrades, sufficiently proves, that the spasmodic cholera of India is neither propagated by contact with the diseased person, nor by the exhalations from his body.

### Fifth Observation.

*On the Sporadic Form of Spasmodic Cholera.*—A case of cholera may now and then be met with, in the healthiest season and situation, in which the disease frequently assumes its worst form, although the patient had, to all appearance, previously evinced no predisposition towards it. Are we here presented with an instance of the development of a latent impregnation? or must we admit that, by the local existence of the choleric miasm, an individual, to all human appearance in the most perfect health, may be taken in a very few hours fiom among thousands of lookers-on, who, amidst the delights of the healthy winter season of India, bury and forget the disease and its solitary victim!

### Sixth Observation.

*On the Premonitory Signs of Spasmodic Cholera.*—There exists, in some few instances, for several hours, or even for some days, before the attack of collapsed cholera, a dark crescent under the eyes, and a shrunken aspect of countenance; but of upwards of a thousand men, with countenances approaching to anxiety, low spirits, without pain or sickness, or with little of either, detained a day in hospital for observation, during a late choleric season, our author does not remember that, in any one of the number, these symptoms terminated in spasmodic cholera.

### Seventh Observation.

*On the Symptoms of Spasmodic Cholera.*—The disease generally makes its attack suddenly, with great prostration of strength and purging of turbid greenish water, attended or quickly followed by vomiting of a whey-coloured fluid, these evacuations, in the first instance, containing portions of the natural contents of the primæ viæ. Spasms of the extremities and universal collapsion quickly succeed; the eyes lose their lustre, sink deep in the sockets, and are surrounded, in the direction of the orbicularis, by a livid circle; the skin contracts over the muscles which are universally shrunken; the countenance assumes a ghastly expression; the tongue is slimy or dry, and soon becomes cold; the thirst is intolerable and cannot be quenched; the urine is suppressed; the hands are blanched, shrivelled, cold, and clammy; the pulse threadlike, irregular, soon becoming imperceptible at the wrists and temples; and the restlessness is indeed terrible to bear. After an uncertain time the spasms and evacuations cease, the breathing becomes hurried, the mind loses its equilibrium, and increased restlessness or insensibility lead to the final catastrophe, generally in a very few hours after the attack.

A milder form of Cholera is ushered in by the symptoms common to inflammatory fever, with severe cramps of the extremities, after reaching the viscera of the abdomen. Vomiting and purging generally accompany the attack, which is also attended with a sense of burning pain in the stomach. In this variety of the disease, the collapse is less urgent, reaction more frequently takes place, and the patient either recovers or lingers several days.

### Eighth Observation.

*On the Treatment of Spasmodic Cholera.*—An endeavour to throw even a little light upon this dark and cheerless subject, requires that it be considered under the varieties which were noticed in the last observation, namely, cholera with collapsion and cholera with excitement—for although the generalizing plan of treatment has, in theory at least, become so common, that we have heard one authority pronounce bleeding the only remedy for cholera, and another declare that there is nothing to be depended on but brandy and laudanum, yet we cannot force indiscriminately into practice agents so opposite to each other in their effects, without feeling that we are abandoning the laws of scientific medicine, and plunging into a chaos of empiricism where pathological reasoning never entered.

In the collapsed cholera, the stomach and bowels have been unloaded of their natural contents before the physician arrives, and sunken features, cold, bedewed skin, cramps of the limbs, cold tongue, intense thirst, scarce perceptible pulse, vomiting and purging of whey-like liquid, and universal prostration, are the prominent symptoms.

The indications of cure in such a state appear to be, to subdue the excessive nervous irritability, and to restore the circulation to its healthy standard.

We must deplore that practical experience has yet found nothing more powerful, in fulfilling these indications, than the means which we are about to enumerate, viz. the combination of calomel and opium, in the proportion of twenty grains of the former with four grains of the latter, administered as early as possible, and repeated according to the urgency of the symptoms and the frequency of its rejection by vomiting; sulphuric æther and aromatic spirit of ammonia, in large and repeated doses, with camphorated emulsion; magnesia suspended in any stimulating aromatic water; and brandy, wine, and other stimulants of that class, in sago gruel. Clysters containing the turpentine and castor oils, with or without assafœtida or opium. Blisters from hot water, cantharides, or mustard, applied to the abdomen, spine, or soles of the feet; a continued warm stimulating friction over the surface of the limbs; and warm bricks applied to the extremities and to the hypochondriac regions. If the irritability of the stomach subsides under this treatment, calomel and colocynth, castor oil and tincture of jalap, or any other cathartic which has the power of accelerating the flow of bile into the intestines, is administered until the excretions resume the feculent and bilious appearance, when recovery usually takes place under the use of light aperient tonics, and the common attention to diet and regimen.

In the febrile form of cholera we are generally presented with a robust person writhing in the agonies of violent tonic spasm especially severe in the calves of the legs. The patient's countenance is flushed and desperate; his pulse is full and frequent, and vomiting and purging are often, but not always present. To hesitate here whether the abstraction of blood, or the administration of stimuli is the better practice, is, in all probability, to sacrifice the patient, for happily this form of cholera is more amenable to the remedial means which we possess. Blood is drawn until the spasms relax, or the excitement gives way, and the recovery is promoted, by exciting the action of the liver and allaying the inordinate irritability of the system. If however collapsion supervenes, stimulant and antispasmodic means are employed, as if the disease was in its first stage.

There is yet another state in spasmodic cholera, where the abstraction of blood becomes indispensible, namely, when the efforts of Nature and the powerful stimulants which have been employed to excite or assist these efforts, bring on a reaction in the system which endangers the safety of the brain. In this case a strict antiphlogistic plan of treatment is necessary, keeping in mind, however, that the vital powers have been artificially excited, that the reviving constitution will not bear much depletion, and that the oppressed brain will probably be more effectually relieved by taking blood from the head, than by large bleedings from the arm.

It would be superfluous to enumerate the palliatives which are usually employed in the last stage of cholera, to alleviate the more distressing sufferings, and smooth the passage to the grave; but we must not pass over in silence the claims of two remedial agents to a more extensive trial than they have yet met with in this disease, viz. the inhalation of diluted oxygen, or nitrous oxyd gas; and the application of the vapour of subliming cinnabar to the surface of the body.

The author concludes this observation on the treatment of cholera with the remark, that the already too wide field of practice, in this disease, is extending, is yet untrod by a master, and offers to the physician who may be called to labour in it, materials in one short season for an age of reflection.

### NINTH OBSERVATION.

*On the Appearances after Death in Spasmodic Cholera.* Scarce has the victim of cholera ceased to breathe, in India, when the livor of decomposition begins to make its appearance, and at six hours after death dissection generally presents the internal parts of the body in the following state. The vessels of the cerebral envelope are turgid with dark-coloured blood; the brain is firm and sound; its vessels also are more or less loaded. The lungs are sometimes inflated, at other times collapsed, their blood-vessels are full of dark-coloured blood, and their minute vascular structure is blanched with water; the coronary vessels are gorged with dark liquid blood, and the heart and large blood-vessels contain more or less of the same fluid. The liver appears of various sizes and colours in different subjects; its vessels are full of dark incrassated blood, and the gall-bladder is distended with viscid bile. The villous coat of the stomach is more or less suffused with a slight erethematic blush, especially about the cardiac orifice; this viscus contains a quantity of whitish turbid water, with a fatty scum, and in the most rapid cases food and medicine, on which digestion had made no impression. The inner surface of the small intestines exhibits also a slight inflammatory tinge, and the whole tube contains more or less of the peculiar wheyish matter, with a viscid pulp so adherent to the inner surface, that it seems to be a partial solution of the villous coat. The spleen appears to undergo but little change during the attack of cholera; in instances it is sound, in others converted by chronic disease, into an almost inorganic mass. The urinary bladder is empty and contracted; and the muscular structure of the body is firmer and darker-coloured than in the healthy state.

The author is of opinion that the congestion in the encephalic vessels for the most part immediately precedes and produces death in cholera.

We have given the substance of Dr. Smith's paper almost entirely in his words, since the language could not be condensed, and the matter appears valuable.—ED.

